# Sanitary Science: Past and Present

**Published:** 1858-10

**Authors:** 


### SANITARY SCIENCE, PAST AND PRESENT.*

A LITTLE book, very unassuming in its externals, lies before us for review. It bears the title given above; and it formed, originally, the annual address delivered before the British Medical Association in 1857. We have rarely seen so much knowledge compressed into so small a compass.

Going far back into the history of the world, Dr. Robertson, the author of this address, sketches out the hygienic codes of Moses, Lycurgus, and Hippocrates: the great ancient sanitary trio. Thence, he glances at the labours of Galen, Oribasius, Aëtius, Paulus Ægineta, and closes the list of sanitarians of the classic age.

Touching briefly on the revival of sanitary teaching at Salerno, and on the writings of Bacon and Sanctorius, he brings us to later days and later names,—to Sydenham, Ramazzini, Pringle, Tissot, Frank, Michaelis, Zuckert, and others still more modern. Changing then his matter of discourse, Dr. Robertson describes what is now being done in different countries in the progress of the sanitary movement. His descriptions on this point are so useful and accurate, that we transcribe them at length:

*Hygiene in France.* "The first sanitary police in France was established about A.D. 1350, and this has been considered the commencement of sanitary reform. During the fifteenth and sixteenth centuries, various ordinances were enacted by which obnoxious trades were banished beyond the boundaries of Paris; but the first council of health in that city was established in 1802, and has

---

* Sanitary Science; its Past and Present State. By W. TINDAL ROBERTSON, M.D. London: Walton and Maberley. 1858.

been in full operation to the present time. It consists of twenty-six members including its officers; and though it is not a legislative body, it is always consulted in matters of hygiene, and its decisions are almost invariably carried out. Reports (amounting in number, up to the end of 1845, to 7,518) are published and addressed to the administration. They are included in three great divisions,—*health*, *salubrity*, and *industry;* and under one or other of these definitions the minor branches of hygienic investigation are comprised.

"Similar councils to those of Paris were established before 1830, in the principal provincial towns; and in 1848, a law providing for a general health ordinance was promulgated by the council of state, and brought into general operation.

"By this law, it is enacted that each *arrondissement* (a district somewhat similar to an English county) should elect a council of health, varying in number from seven to fifteen persons. The members hold office for four years, and they are charged with the examination of all subjects relative to public health which may be submitted to them by the prefect or sub-prefects of the districts. Besides these duties, they must issue reports on medical statistics, topography, and charitable institutions, and the reports for the district are condensed and classified by the councils of the departments before being finally submitted to the minister who has special charge of the health department."

*Hygiene in Germany.* "In the German states, general hygiene has been more extensively applied to society than in any other country. The system consists in a series of subordinate bureaux in direct connection with the home-secretary by means of a central medical section. The director of this section acts as an under-secretary of state; and it is composed of three physicians, two apothecaries, and two veterinary surgeons. In addition to this, there is a central council of health, composed of eminent medical practitioners, who advise the administration on all questions of medical science and hygiene. Reporting to the central board, are provincial boards (*Landes Collegien*), superintended by a government medical counsellor (*Regierungs medicinalrath*), and subordinate to these, are the districts, 335 in number, superintended by the *Kreis-physici.*

"The *Kreis-physicus* must be a surgical physician, and pass an examination in state-medicine, and one in veterinary state-medicine. He is assisted by a district surgeon (*Kreis-wundartz*), and his duties are important and multifarious. Amongst them are the following,—quarterly reports on meteorology, state of the crops, prevailing epidemics, and the veterinary practice of the quarter. The JOURNAL OF PUBLIC HEALTH has lately instituted similar reports from observers in different parts of this country, the object being to institute comparisons between one district and another, and to collect information on these important subjects. In addition to the quarterly reports, a yearly report on the state of the profession in the province is required, and medical topography, hospitals, baths, schools, *post mortem* examinations, and a subject which is only just beginning to receive attention here, I mean the collection of agricultural statistics, are all entrusted to the same agency.

"I have been describing the system as it exists in Prussia, and although there is in each German state a difference in points of detail, yet, in each the main features are similar to those which have been mentioned. In Hesse, there is a Kreis-medicinal Rath, or Provincial Physicus, for each district containing 40,000 inhabitants. He is a sort of medical rural-dean, or medical councillor, and subordinate to him are the Physikatsärtze, or officers of health. In Nassau, no physicians practise privately. The whole population is divided into districts, to each of which there is attached a medicinal rath, who is supplied with an assistant and a junior assistant."

*Hygiene in America.* "The first Board of Health in America was established at Boston in 1799. This was modified in 1816, and the two statutes by which these were constituted have served for models, after which succeeding ones have been formed. Under their provisions, the following officers are appointed:—

I. A Superintendent of Streets.
II. A Superintendent of Common Drains and Sewers.
III. A Water Board.
IV. A City Physician, to examine into nuisances, &c.
V. A Port Physician, to superintend quarantine.
VI. Five Consulting Physicians, in case of the breaking out of any dangerous disease
VII. A City Registrar.
VIII. A City Marshal to act as General Health Officer.

"Great improvements are contemplated; and a commission which was appointed in 1849, to make a sanitary survey of the state, has issued an elaborate and most instructive report, in which a series of measures of importance are proposed, and the necessity for their adoption urged with the greatest energy and effect. Amongst other things, they recognise hygiene as a distinct branch of education. In a report by the Hon. H. Mann, one of the most intelligent writers who have ever taken pen in hand for the service of their country, occurs the following passage:—'All the teachers just out of our normal schools are well grounded in the elements of human physiology. *They introduce it wherever they go.* It has been prominent at all our teachers' institutes. I have lectured upon it hundreds of times, to teachers and school associations, and I estimate that no less than 15,000 annually of the children in our public schools attend to this subject well.'

"The Public Health Act, passed by Congress, April 24th, 1850, provides, *inter alia*:—

"Sect. 1. 'Physiology and hygiene shall hereafter be taught in all the public schools of this commonwealth, in all cases in which the school committee shall think it expedient.'

"Sect. 2. 'All school teachers shall hereafter be examined in their knowledge of the elementary principles of hygiene and physiology, and their ability to give instructions in the same.'

"I come now, after this brief and imperfect sketch of the state of hygiene in other nations, to that in which we are more immediately interested, that of our own country."

From abroad, Dr. Robertson naturally travels home, and having dwelt on Lord Morpeth's Act and explained its provisions, he closes his instructive paper with suggestions as to what has to be performed in the future.

Accustomed to labours somewhat akin to those undertaken by our author, we can appreciate in some degree the amount of research which lies, not on the surface, but at the foundation of his little book, which is, in fact, a most unfair account "of work and labour done". We therefore suggest, that Dr. Robertson let this his present work stand as the mere syllabus of a greater history of sanitary science; a work which, well done, is a desideratum, and would establish his literary fame.

---

